# 磁性碳凝胶磁固相萃取与液相色谱联用分析矿泉水和泡面碗中4种溴代阻燃剂

**DOI:** 10.3724/SP.J.1123.2024.08005

**Published:** 2025-06-08

**Authors:** Qinrong NIE, Ming NI, Jiangyan XU, Ying SHI, Hongmei JIANG

**Affiliations:** 南京农业大学理学院，江苏 南京 210095; College of Science，Nanjing Agricultural University，Nanjing 210095，China

**Keywords:** 磁性碳凝胶, 磁固相萃取, 高效液相色谱, 溴代阻燃剂, 矿泉水, 泡面碗, magnetic carbon aerogel, magnetic solid-phase extraction （MSPE）, high performance liquid chromatography （HPLC）, brominated flame retardants （BFRs）, mineral water, instant-noodle-bowl

## Abstract

作为塑料制品中应用极广的有机阻燃剂，溴代阻燃剂大多具有较强的生物毒性和稳定的理化性质，通过间接或直接与食品接触，不可避免地残留在日常食物中，为人类健康带来潜在危害。因此，迫切需要建立一种快捷有效的溴代阻燃剂分析方法。磁固相萃取法因具有操作简单、可快速磁分离等优点，在痕量分析中得到了广泛的应用，该方法的关键在于高效磁性吸附剂的研制。本文提出以溶胶凝胶法结合煅烧法制备磁性碳凝胶，并以其为磁固相萃取材料，建立了磁固相萃取与高效液相色谱联用分析矿泉水和泡面碗中4种溴代阻燃剂的新方法。采用傅里叶变换红外光谱、X-射线衍射以及透射电镜等技术对材料的结构和组成进行表征，证实了磁性碳凝胶的成功制备。对影响磁固相萃取的因素如溶液pH值、材料用量、吸附时间、洗脱溶剂的浓度与体积和样品体积进行了详细考察，在最优条件下，本方法对四溴双酚A、3-溴联苯、4，4′-二溴联苯和四溴联苯醚的检出限（*S/N*≥3）分别为0.005、0.005、0.005和0.010 mg/L；RSD分别为7.35%、5.12%、3.66%和5.58%（*n=*5，*C*=0.02 mg/L）；实际富集倍数分别为50、40、51和61倍。最后将所提出的方法应用于农夫山泉水样和塑料泡面碗中4种溴代阻燃剂的测定，获得了满意的加标回收结果，为溴代阻燃剂的分析提供了一种快捷、灵敏的新方法。

## Abstract (en)

Brominated flame retardants （BFRs） are widely used as organic flame retardants in plastic products， with most exhibiting strong biological toxicity as well as physical and chemical stability. BFRs inevitably remain in foods consumed on a daily basis through indirect or direct contact， thereby threatening human health. Therefore， establishing a fast and effective method for detecting and analyzing BFRs is imperative. Magnetic solid-phase extraction （MSPE） has been widely used in trace-analysis applications owing to advantages that include operational simplicity and rapid magnetic separability. The key to MSPE lies in the design and preparation of efficient magnetic adsorbents. In this study， a magnetic carbon aerogel （MCA） was prepared using a sol-gel method in combination with calcination. MCA was used as a magnetic solid-phase extractant to establish a new method for the analysis of four BFRs in mineral water and instant-noodle-bowl samples in combination with high performance liquid chromatography. Fourier-transform infrared （FT-IR） spectroscopy revealed peaks at 3 454， 1 590， 757， 1 349， 1 654， and 1 076 cm^-1^ that are ascribable to -NH_2_， -CH， triazine-ring， C-N， C=N， and C-O-C vibrations， respectively. Brunauer-Emmett-Teller （BET） analysis revealed values of 192.16 m^2^/g， 0.34 cm^3^/g， and 7.12 nm for the surface area， pore volume and pore size of the MCA， respectively. X-ray diffractometry （XRD） revealed a characteristic peak at 2*θ=*34.90° that corresponds to the （110） crystal plane of Fe_2_O_3_， and peaks at 2*θ* values of 44.72°， 65.01° and 82.42° that are ascribable to the （110）， （200）， and （211） crystal planes of CoFe/Co_3_Fe_7_. Vibrating sample magnetometry showed that the MCA is highly magnetic （35 emu/g）， which contributes to fast magnetic solid-liquid separation. The MCA was characterized by transmission electron microscopy （TEM）， which revealed a transparent gauze-like structure with nanometer-sized squares and circular particles evenly distributed between them. High-resolution TEM （HRTEM） showed that the square particles exhibit a 0.191 nm stripe spacing that belongs to the （311） crystal plane of Fe_2_O_3_， while the 0.245 nm stripe spacing observed for the circular particles corresponds to the （110） crystal plane of the CoFe alloy， in good agreement with the XRD results. X-ray photoelectron spectroscopy （XPS） revealed the presence of Co 2*p*， Fe 2*p*， O 1*s*， N 1*s*， and C 1*s* peaks. Taken together， these results show that the MCA， which contains various functional groups， had been successfully prepared. Factors that affect MSPE， such as solution pH， amount of material， adsorption time， the concentration and volume of the elution solvent， and sample volume， were investigated using the static adsorption method. BFR adsorption by the MCA was observed to increase with time， with equilibrium eventually reached. Tetrabromobisphenol A （TBBPA） reached adsorption equilibrium at 1 h with an adsorption rate close to 100%， whereas 3-bromobiphenyl （PBB-2）， 4，4′-dibromobiphenyl （PBB-15）， and 2，2′，4，4′-tetrabromodiphenyl ether （BDE-47） reached adsorption equilibria at 2 h. BDE-47 exhibited an adsorption rate close to 80% when 20 mg/L MCA was used， whereas the remaining three BFRs exhibited values close to 100%. Absorption by the MCA initially exhibited a constant trend with increasing sample volume but began to decline as the sample volume exceeded 100 mL. BFR adsorption by the MCA was found to be almost pH-independent， which indicates that the MCA is stable over a wide pH range. In addition， the analytes were effectively eluted in 30 min using 5 mL of acetonitrile. Based on the results presented above， the optimal adsorption and desorption conditions are： 20 mg/L of adsorbent， a sample volume of 100 mL， an adsorption time of 2 h without pH adjustment， 5 mL of acetonitrile as the eluent， and desorption time of 30 min. TBBPA， PBB-2， and PBB-15 exhibited limits of detection （LODs， *S/N*≥3） of 0.005 mg/L each under the optimal conditions， while BDE-47 exhibited a value of 0.010 mg/L， with corresponding RSDs of 7.35%， 5.12%， 3.66%， and 5.58% （*n*=5， *C*=0.02 mg/L）， respectively， and actual enrichment times of 50， 40， 51， and 61 min， respectively. The developed method was used to determine four BFRs in mineral water and instant-noodle-bowl samples， with satisfactory recoveries obtained， thereby providing a new fast and sensitive method for the analysis of brominated flame retardants.

溴代阻燃剂（brominated flame retardants，BFRs）作为目前世界上产量和用量最大的有机阻燃剂，从20世纪70年代开始被广泛应用于塑料、纺织品、电子元件等各类材料中以提升产品的阻燃性^［1］^。由于BFRs具有明显的生物毒性、较强的生物蓄积性、可长距离迁移和理化性质稳定等特点，一些经典的溴代阻燃剂如六溴环十二烷、多溴二苯醚等被《斯德哥尔摩公约》列为持久性有机污染物（persistent organic pollutants， POPs）^［2］^，欧盟也将多种溴代阻燃剂列为优先控制的化学品名单。日常生活中，BFRs被广泛用于各种塑料食品包装材料，与食品直接或间接接触，对食品安全和生物健康带来一定的安全隐患；其中，四溴双酚A （tetrabromobisphenol A， TBBPA）、3-溴联苯（3-bromobiphenyl， PBB-2）、4，4′-二溴联苯（4，4′-dibromobiphenyl， PBB-15）和四溴联苯醚（2，2′，4，4′-tetrabromodiphenyl ether， BDE-47）是4种典型的常用溴代阻燃剂，其化学结构式如图1所示。因此，建立一种快捷有效的BFRs分析方法对减少溴代阻燃剂残留和保障人体健康具有重要的理论和现实意义。目前，针对食品中的溴代阻燃剂已发展出多种分析检测方法，如质谱法（MS）^［1］^、气相色谱法（GC）^［3］^、高效液相色谱法（HPLC）^［4］^等。HPLC具有灵敏度高、检出限低、操作简便等优点，目前已被广泛应用于食品中痕量污染物的检测。

**图1:**
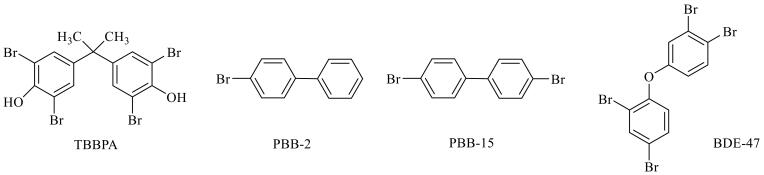
4种溴代阻燃剂的化学结构式

实际样品中BFRs的含量极低且存在大量复杂基体，为了提高分析灵敏度、降低基体影响，测定前需对样品进行分离和预富集。目前，已经发展出了多种较为常用的萃取分离富集方式，如固相萃取（solid-phase extraction，SPE）^［5］^、分散固相萃取（dispersed solid-phase extraction，DSPE）^［6］^、固相微萃取（solid-phase microextraction，SPME）^［7］^、搅拌棒吸附微萃取（stir bar sorptive extraction， SBSE）^［8］^、毛细管微萃取（capillary microextraction， CME）^［9］^、液相微萃取（liquid phase microextraction，LPME）^［10］^、磁固相萃取（magnetic-solid-phase extraction， MSPE）^［11］^等。MSPE因具有分离效率高、有机试剂消耗少、操作简单、可快速磁分离等优点，在污染物分析中得到了广泛的应用^［12，13］^。MSPE应用和发展的关键在于选择高效的磁性吸附剂。常用的MSPE吸附剂有磁性生物材料^［14‒16］^、磁性有机小分子^［17‒20］^、磁性有机高分子^［21‒23］^、磁性金属有机骨架化合物^［24］^、磁性共价有机骨架化合物^［25］^、磁性碳材料等^［26，27］^。这些材料各自具有不同的特点，被应用于不同的分离富集体系。其中，磁性碳材料具有孔隙发达、比表面积大、吸附容量高等优点，在食品分析样品前处理中得到了广泛的应用。

常见的用于磁性纳米粒子改性的碳材料有石墨烯^［28］^、碳纳米管^［29］^、介孔碳^［30］^、生物质炭^［31］^、碳凝胶^［32］^等。磁性碳凝胶（magnetic carbon aerogel，MCA）是一种由溶胶凝胶法合成的三维多孔磁性碳材料，既具有碳凝胶材料大比表面积特性，又兼有磁性材料可快速磁分离的特点。本文提出采用溶胶凝胶法结合煅烧法制备MCA，通过一系列表征技术分析MCA的表面形貌和组成结构，并以其为MSPE材料，将MSPE与HPLC联用，对所研究的MSPE分析性能进行评估，建立一种快捷高效、绿色经济的BFRs分析方法，为经济高效BFRs分析方法的开发提供理论依据。

## 1 实验部分

### 1.1 仪器、试剂与材料

Waters 1525 高效液相色谱仪（美国Waters公司）；Thermo Nicolet 300傅里叶红外光谱仪（Fourier transform infrared spectrometer， FT-IR，美国Thermo Nicolet公司）；V Sorb 2800P比表面积及孔径分析仪（Brunauer-Emmett-Teller， BET，北京金埃谱科技有限公司）；D8advance X射线衍射仪（X-ray powder diffractometer， XRD，德国Bruker公司）；Squid-vsm振动样品磁强计（vibrating sample magnetometer， VSM，美国Quantatech公司）；250xi X射线光电子能谱仪（X-ray photoelectron spectroscopy， XPS，美国Thermo公司）；IS-RDD3台式恒温振荡器（美国Crystal Technology & Industries公司）；ZGDCY-12干式氮吹仪（上海梓桂仪器有限公司）；OTF-1200X真空管式炉（合肥科晶材料技术有限公司）。

溴代阻燃剂BFRs标准品：TBBPA（纯度≥98%）和BDE-47（纯度≥95%）购自上海源叶生物科技有限公司，PBB-2（纯度≥97%）和PBB-15（纯度≥99%）购自北京百灵威科技有限公司。九水合硝酸铁（分析纯）购自国药集团化学试剂有限公司。六水合硝酸钴（分析纯）、三聚氰胺（纯度≥99%）、乙腈（纯度≥99.9%）、环己烷（纯度≥99.5%）、异辛烷（纯度≥99%）和氢氧化钠（分析纯）购自上海麦克林生化科技股份有限公司。甲醛（分析纯）购自广东光华科技股份有限公司。丙酮（分析纯）购自永华化学股份有限公司。甲醇（分析纯）购自美国TEDIA公司。盐酸（优级纯）购自南京化学试剂股份有限公司。

矿泉水和泡面碗为市售。

### 1.2 材料制备

取1.261 2 g（0.01 mol）三聚氰胺与7.5 mL质量分数为37%的甲醛溶液（0.10 mol）加热溶解在40 mL的去离子水中，80 ℃下磁力搅拌成无色透明液体。然后加入浓NaOH溶液，调节溶液的pH为9左右，室温下预聚30 min。接着将温度调节至50 ℃，加入浓盐酸调节pH至1.5左右，继续反应1 h。加入0.436 6 g （0.001 5 mol） Co（NO_3_）_2_·6H_2_O和0.606 0 g （0.001 5 mol） Fe（NO_3_）_3_·9H_2_O金属无机盐，溶解后转移到螺口玻璃瓶中，90 ℃下老化一定时间，得到红棕色的水凝胶。将水凝胶浸没于丙酮中进行溶剂置换，该操作重复两次，每次12 h。接着将凝胶进行干燥处理，得到干凝胶。最后将干凝胶置于管式炉中，在氮气气氛下700 ℃煅烧2 h，得到所需MCA。煅烧后的材料研磨，待用。

### 1.3 磁固相萃取过程

将20 mg MCA置于100 mL干净烧杯中，加入一定浓度的溴代阻燃剂溶液，采用保鲜膜将烧杯密封后，置于台式恒温振荡器中振荡2 h，取上清液用滤膜过滤后使用HPLC检测残液中4种溴代阻燃剂浓度，计算吸附率；将吸附后材料取出，置于烧杯中，加入5 mL乙腈，振荡30 min，取洗脱液1 mL，用0.22 μm滤膜过滤至1.5 mL离心管中，室温下氮吹浓缩为0.2 mL，采用HPLC检测4种溴代阻燃剂的质量浓度，计算脱附率。吸附率和脱附率计算公式见式（1）和式（2）。

吸附率=(*C*_0_-*C*_e_)/*C*_0_×100% （1）

*D*=*m*_d_/*m*_0_×100% （2）

其中*C*_0_（mg/L）是吸附前各溴代阻燃剂的初始质量浓度，*C*_e_（mg/L）是吸附平衡后残液中溴代阻燃剂的质量浓度。*D*是脱附率，*m*_d_是解吸溶液中溴代阻燃剂的质量，*m*_0_是MCA上吸附的溴代阻燃剂的质量；*m*_d_可由解吸后目标分析物的浓度与脱附体积的乘积求得，*m*_0_由溴代阻燃剂初始质量浓度与吸附平衡后残液中质量浓度之差、再与样品体积的乘积求得。磁固相萃取流程图如图2所示。

**图2:**
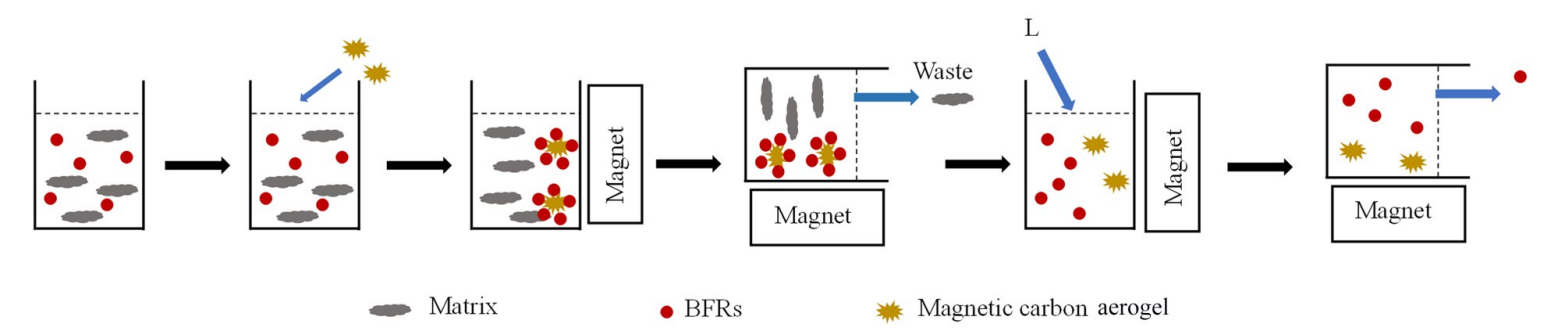
磁固相萃取流程图

### 1.4 色谱条件

色谱柱：Agilent 5 TC-C18 （2） （250 mm×4.6 mm，5 µm）。流速：1 mL/min。进样量：20 μL。流动相：A相-Na_2_HPO_4_/KH_2_PO_4_缓冲溶液，B相-甲醇。洗脱条件：初始条件为95%B，0~5 min线性变化至100%B，5~8 min保持100%B。

### 1.5 实际样品处理

对所选取的矿泉水样品，使用孔径为0.45 μm的水系滤膜进行过滤，收集滤液待用。

对所选用的泡面碗样品，采用自来水、去离子水清洗后，分别使用乙醇、甲醇进行洗涤，室温下风干后剪成直径不超过2 mm的细小颗粒，备用。准确称取各样品1.00 g于100 mL带塞锥形瓶中，并加入5 mL乙腈作为提取剂，超声提取30 min后抽滤过滤。滤渣复提一次，将两次滤液合并到离心管中，氮吹至1 mL。将所得1 mL提取液用超纯水溶解，定容至100 mL带塞锥形瓶中，待用。

## 2 结果与讨论

### 2.1 材料的表征

#### 2.1.1 FT-IR分析

对MCA进行FT-IR分析，结果如图3所示，可以得出MCA的主要官能团结构。其中，3 454 cm^‒1^处的宽峰对应氨基（-NH_2_）和羟基（-OH）的伸缩振动。2 850~2 950 cm^‒1^处的小峰对应-CH伸缩振动。位于1 590 cm^‒1^与757 cm^‒1^处的峰则共同说明材料存在三嗪环^［33］^，这一官能团直接来自原材料三聚氰胺。在1 349 cm^‒1^处的峰归属于碳氮键（C-N），1 654 cm^‒1^处的峰则来自氨基的弯曲振动和碳氮双键（C=N）的伸缩振动。1 076 cm^‒1^处的峰来自醚键（C-O-C）的伸缩振动，这证明三聚氰胺与甲醛交联形成了醚桥，表明三聚氰胺甲醛凝胶的形成^［34］^。

**图3:**
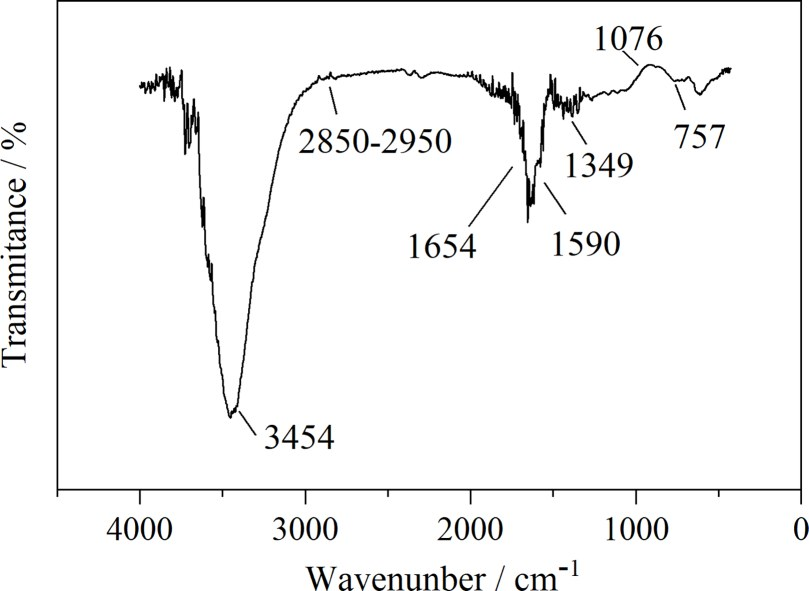
磁性复合材料MCA的红外图

#### 2.1.2 BET分析

对MCA的比表面积进行了分析表征，结果表明材料具有较大的比表面积（192.16 m^2^/g）和孔体积（0.34 cm^3^/g），平均孔径为7.12 nm。从图4可以看出材料的吸脱附氮气曲线为含有H3型介孔回滞环的Ⅳ型等温线。H3型介孔回滞环说明MCA含有不规则的狭窄裂隙孔结构，Ⅳ型等温线则说明材料对氮气的吸附是发生在介孔壁上的单层-多层吸附。

**图4:**
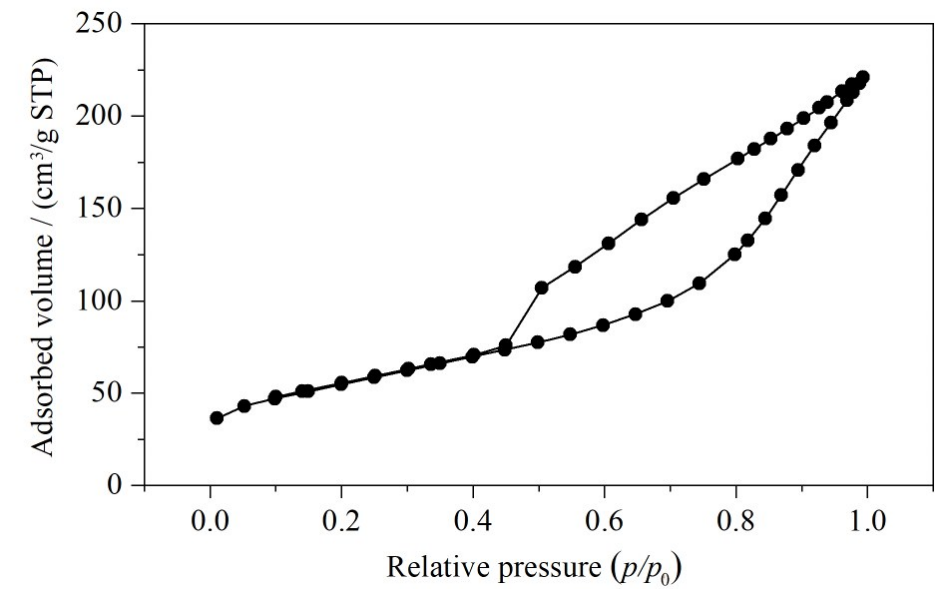
复合材料MCA的氮气吸附脱附等温线

#### 2.1.3 XRD分析

对MCA进行了XRD分析，确定了材料的晶体结构和物相组成，结果见图5。

**图5:**
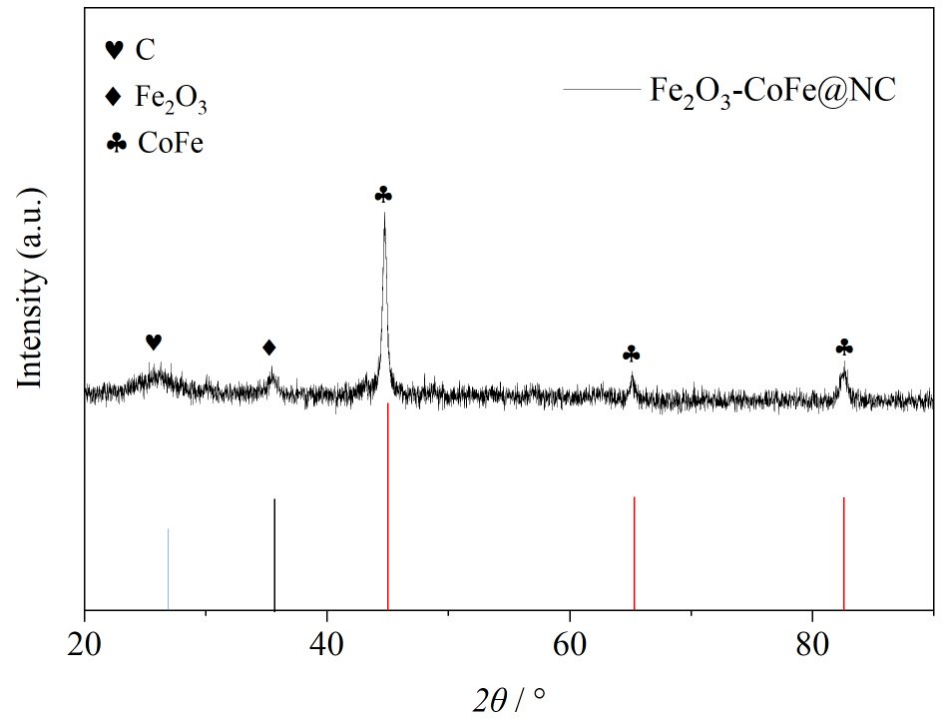
复合材料MCA的XRD图

从图5中可以观察到明显的峰位。图中2*θ*=26.60°处的衍射峰对应于石墨化碳的（002）晶面，2*θ*=34.90°处的衍射峰归属于Fe_2_O_3_的（110）晶面，而2*θ*=44.72°、65.01°和82.42° 3处的衍射峰分别归属于CoFe/Co_3_Fe_7_的（110）、（200）和（211）晶面。这些结果说明MCA中金属元素的可能存在形式为三氧化二铁和钴铁合金。

#### 2.1.4 VSM分析

采用振动样品磁强计分析了MCA的磁性能，结果见图6。可以发现，MCA饱和磁矩约为35 emu/g，说明该材料具有较强的磁性，在外加磁场作用下可很好地进行固液分离。

**图6:**
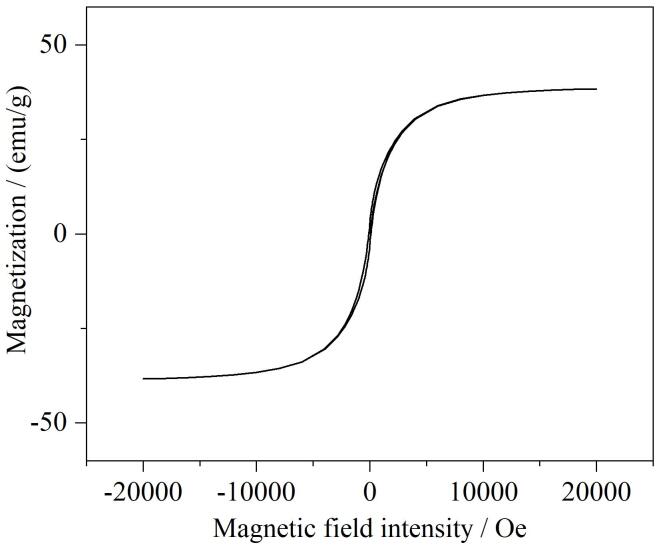
复合材料MCA的磁滞曲线

#### 2.1.5 TEM分析

对MCA进行了透射电镜分析，结果如图7所示。可以看出，MCA呈现透明的薄纱状，推断为石墨化的氮碳，其间较为均匀地分布着一些纳米级别的方形和圆形颗粒，可能为金属氧化物和合金颗粒。MCA的高分辨透射电镜结果（图7b和7c）显示出明显的晶格条纹，方形颗粒中0.191 nm的条纹间距归属于Fe_2_O_3_的（311）晶面，圆形颗粒中0.245 nm条纹间距对应CoFe的（110）晶面，证明了方形和圆形颗粒分别对应于Fe_2_O_3_和CoFe合金，这些结果与X射线衍射结果吻合，进一步证实了材料的组成。

**图7:**
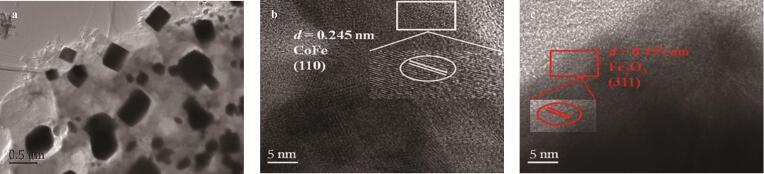
MCA的（a）透射电镜图谱和（b，c）高分辨透射电镜图谱

#### 2.1.6 XPS分析

对MCA进行了XPS分析，结果如图8所示。从图8a中可以看出，MCA的总谱包含钴、铁、碳、氧和氮5种元素，与XRD显示的物相组成元素吻合。由图8b可以看出，781.25 eV和796.86 eV处的峰分别为Co_2_ *_p_*_3/2_和Co_2_ *_p_*_1/2_；根据图8c可知，711.09 eV和724.50 eV处的峰分别对应于Fe_2_ *_p_*_3/2_和Fe_2_ *_p_*_31/2_；C分谱（图8d）中284.60、286.45和288.10 eV处的谱峰分别代表石墨化碳、N-C=N以及C=O；N分谱（图8e）中398.5、400.8和403.35 eV处的谱峰分别对应吡啶氮、吡咯氮和石墨化氮；O分谱（图8f）中530.37 eV和531.74 eV处的谱峰分别代表M-O和C=O，532.86 eV处的谱峰则代表C-O和O-H，这些结果说明MCA已被成功制备，且这些官能团有助于对BFRs的吸附保留。

**图8:**
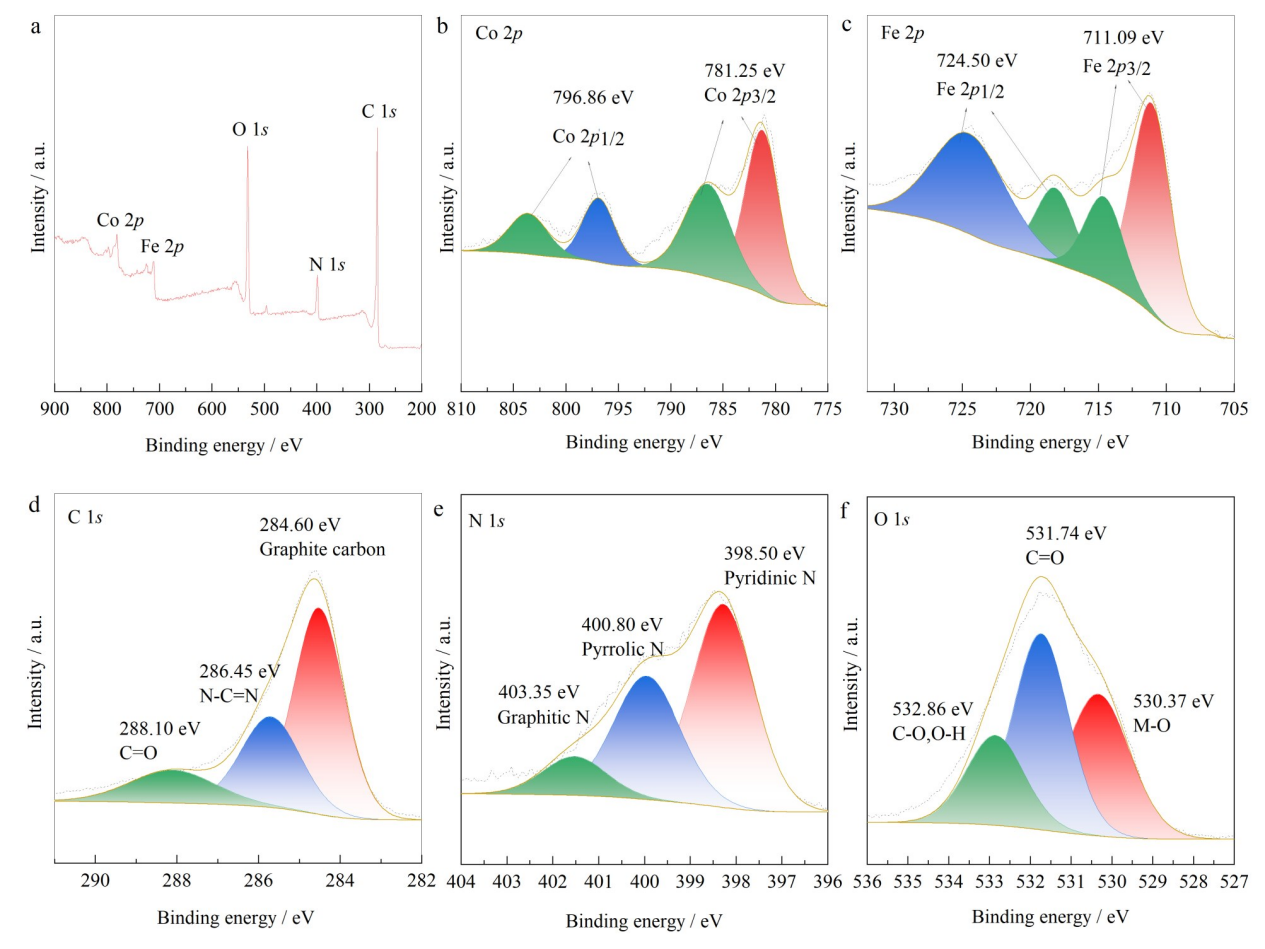
MCA的（a）XPS总谱、（b） Co 2*p*， （c） Fe 2*p*， （d） C 1*s*， （e） N 1*s*和（f） O 1*s*的XPS分谱图

### 2.2 MSPE条件优化

#### 2.2.1 吸附时间

吸附速率决定了材料到达吸附平衡所需时间，本研究考察了吸附时间对MCA吸附溴代阻燃剂的影响，结果如图9a所示。可以看出，MCA对4种溴代阻燃剂的吸附均呈现先增加后平衡的趋势，其中，TBBPA在1 h时达到吸附平衡，吸附率接近100%，PBB-2、PBB-15和BDE-47 3种物质于2 h达到吸附平衡。这是因为在吸附刚开始时，MCA表面存在较多的空白吸附位点，溴代阻燃剂易与空白吸附位点接触，使得吸附率随时间的增加而急剧增大；随着时间的延长，空白吸附位点逐渐被相应的溴代阻燃剂占据，吸附效率增长速度放缓；最后，当达到一定时间时，MCA表面的吸附位点完全被溴代阻燃剂占据，吸附效率达到最大。因此，后续实验我们选择吸附时间为2 h。

**图9:**
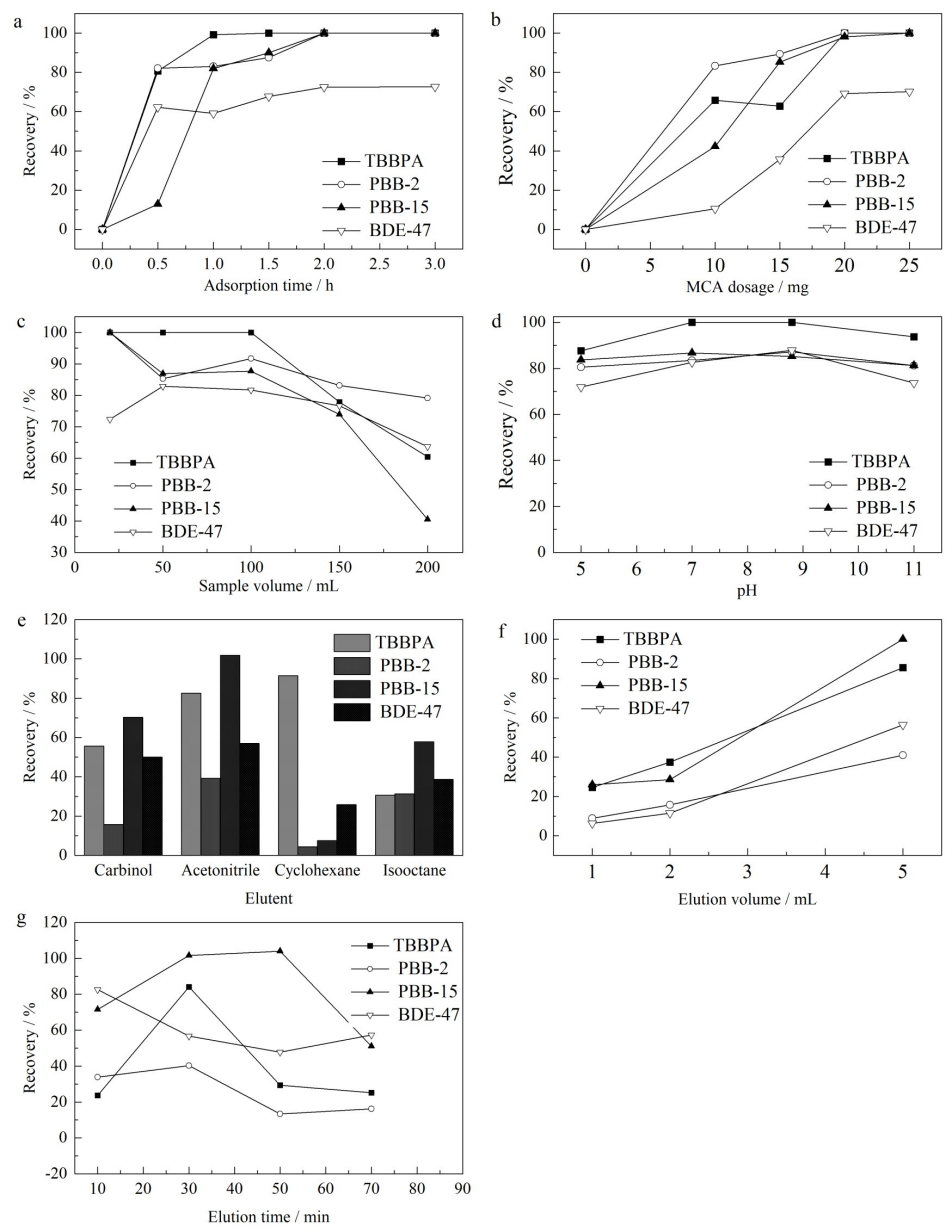
（a）吸附时间、（b）材料用量、（c）样品体积、（d） 样品pH、（e）洗脱剂类型、（f）洗脱剂体积和（g）洗脱时间对4种溴代阻燃剂回收率的影响

#### 2.2.2 材料用量

本研究还考察了吸附剂添加量对4种溴代阻燃剂吸附效率的影响，结果如图9b所示。可以看出，随着吸附剂用量的增加，4种溴代阻燃剂的吸附效率整体呈现先逐步提高，后保持不变的趋势，当MCA添加量达到20 mg时，BDE-47的吸附率接近80%，其余3种溴代阻燃剂均接近100%。这是因为吸附剂用量增加可以提供更多的吸附位点，有助于吸附更多的溴代阻燃剂；随着更多吸附剂的加入，溶液中的溴代阻燃剂大部分都已被吸附至MCA表面，进而达到吸附平衡。后续选取20 mg为最佳吸附剂用量。

#### 2.2.3 样品体积

本研究考察了MCA在不同样品定容体积下对溴代阻燃剂的吸附能力，结果如图9c所示。可以看出，随样品体积的增大，MCA对4种溴代阻燃剂的吸附率均呈现先恒定后下降的趋势。这是因为当样品体积过小时，初始浓度较高，各种溴代阻燃剂吸附驱动力较大，导致MCA对4种溴代阻燃剂均呈现较好的吸附效率。随着样品体积的增加，溶液浓度减小，各目标化合物传质驱动力下降，最终导致吸附率下降。综合4种溴代阻燃剂吸附率的变化趋势，最终选取100 mL的样品体积进行后续实验。

#### 2.2.4 溶液pH值

体系pH值会影响4种溴代阻燃剂的存在形态和吸附剂表面官能团的活性，进而影响吸附剂的吸附行为。本研究探究了pH对MCA吸附4种溴代阻燃剂的影响，结果如图9d所示。可以看出，在所研究的pH范围（5~11）内，MCA对4种溴代阻燃剂的吸附率仅出现微弱的波动，说明MCA可在较广pH跨度内维持稳定，从而有希望被用于广范围、多体系的实际样品分析。这是因为目标化合物溴代阻燃剂多为中性分子，pH值对中性分子的影响微乎其微。因此，后续实验不对初始pH值进行人为调整。

#### 2.2.5 洗脱剂种类

考察了甲醇、乙腈、环己烷和异辛烷对4种溴代阻燃剂的洗脱效果，结果见图9e。环己烷的洗脱效果最差，而乙腈的洗脱效果最佳。这是因为洗脱溶剂对目标成分的解吸附作用通常利用“相似相溶”原理，即相似的化学物质往往能够相互溶解。4种溴代阻燃剂均为极性物质，而乙腈具有良好的溶解性和化学稳定性，且具有较高的极性，这使其可以与溴代阻燃剂发生较强的相互作用，因此能够有效地溶解和洗脱溴代阻燃剂。综上，本文选择乙腈作为洗脱剂。

#### 2.2.6 洗脱剂体积

考察了洗脱剂乙腈体积对4种溴代阻燃剂的洗脱效果，结果见图9f。可以看出，随着乙腈体积的增加，洗脱效率也逐步提升，当乙腈体积为5 mL时，洗脱效果最佳。这是因为洗脱剂用量会对其与吸附剂的接触程度造成影响，一定量的乙腈对目标溴代阻燃剂的溶解能力是有限的，当其达到溶解平衡后会停止对目标化合物的继续洗脱。增加乙腈的体积可扩大洗脱剂对4种溴代阻燃剂的溶解容量，确保洗脱剂与吸附剂充分接触，这有助于乙腈分子更充分地渗透到吸附剂内部，从而更有效地将目标物质从吸附剂上洗脱下来，提高洗脱效率。若继续增加洗脱剂体积，采用10 mL乙腈进行洗脱，则4种溴代阻燃剂无法进行有效分离，从而无法确定洗脱率。故最终选取5 mL乙腈作为洗脱剂。

#### 2.2.7 洗脱时间

洗脱时间可在一定程度上决定溴代阻燃剂从MCA上的解吸效率，本研究考察了不同洗脱时间（10~70 min）对乙腈洗脱4种溴代阻燃剂的影响，结果如图9g所示。对BDE-47而言，洗脱效率随洗脱时间的增加而缓慢降低，而对另外3种溴代阻燃剂，其洗脱率均随时间变化呈现先增加后降低的趋势。在30 min时，TBBPA和PBB-2达到最高洗脱率，而PBB-15的洗脱率峰值出现在50 min时。在洗脱过程的初始阶段，洗脱率通常会随时间的推移而迅速增加，这是因为乙腈与MCA上的溴代阻燃剂之间的浓度差较大，形成了较大的传质推动力，使得溴代阻燃剂能够快速地被解吸。随着洗脱时间的延长，MCA上溴代阻燃剂浓度逐渐降低，乙腈中溴代阻燃剂浓度逐渐增加，导致传质推动力减小，洗脱效率增长速度逐渐放缓。当洗脱时间足够长时，MCA上的大部分溴代阻燃剂已被解吸，剩余的少量溴代阻燃剂由于MCA的强吸附作用或其他因素而难以被解吸，使得洗脱效率会趋于稳定或略有下降。此外，过长时间的洗脱可能使被洗脱的溴代阻燃剂重新被MCA吸附，从而降低洗脱效率。为同时保证4种目标物一定的洗脱效率，最终选择30 min为洗脱时间。

### 2.3 分析性能

本法对4种溴代阻燃剂的分析性能结果如表1所示。可以看出，经MSPE处理后，目标物在0~0.1 mg/L范围内具有良好的线性关系（*R* ^2^=0.998 7~0.999 8）；检出限（LOD， *S/N*=3）为0.005~0.010 mg/L，定量限（LOQ， *S/N*=10）为0.016 5~0.033 mg/L；相对标准偏差分别为7.35%、5.12%、3.66%和5.58%（*n=*5，*C*=0.02 mg/L）；将经MSPE处理后制作的标准曲线与未经前处理的标准曲线线性方程的斜率相比得到各化合物的实际富集倍数^［39］^，分别为50、40、51和61。将本法与文献报道的方法进行比较，结果列于表2，可以看出，本方法灵敏度适中，可用于复杂塑料基体样品中溴代阻燃剂的分析。

**表 1:** 方法的分析性能

Analyte	Liner equation after MSPE ^a^	*R* ^2^	LOD/（mg/L）	LOQ/（mg/L）	RSD/%^b^	Enrichment factor
TBBPA	*Y*=2732719.5*X*+2694.1	0.9998	0.005	0.0165	7.35	50
PBB-2	*Y*=2456021.1*X*-8836.9	0.9987	0.005	0.0165	5.12	40
PBB-15	*Y*=2696181.3*X*+147.4	0.9995	0.005	0.0165	3.66	51
BDE-47	*Y*=3315494.0*X*-8380.1	0.9993	0.010	0.033	5.58	61

a： linear range 0-0.1 mg/L； b： mass concentration 0.02 mg/L， *n*=5. *Y*： peak area； *X*： mass concentration， mg/L.

**表 2:** 本方法与文献报道方法比较

Analysis method	Adsorbent	Analytes	Samples	LOD/（μg/L）	Ref.
SPE-HPLC	Br-COF	polybrominated diphenyl ethers	food	0.01-0.05	［^35^］
MSPE-GC-MS	micro-nano-structured magnetite particles	tetrabromobisphenol A， hexabromocyclododecane	water	130-350	［^36^］
MSPE-HPLC	NiFe_2_O_4_@COF	brominated flame retardants	water	0.03-1.9	［^37^］
MSPE-HPLC	Fe_3_O_4_@COF	brominated flame retardants	water	0.15-0.39	［^38^］
MSPE-HPLC	MCA	brominated flame retardants	water， plastic bowl	5-10	this work

### 2.4 实际样品分析及回收率试验

为了验证该方法的可行性与实际应用价值，将本法直接应用于矿泉水和泡面碗中TBBPA、PBB-2、PBB-15和BDE-47 4种溴代阻燃剂的分析测定。取6份100 mL矿泉水样品的滤液和6份100 mL泡面碗提取液的稀释液，各分为两组，分别加入中、高2个不同水平的标准溶液，按照1.3节方法完成前处理过程，使用HPLC检测目标溴代阻燃剂的浓度并计算回收率。由表3可以看出，加标试验的回收率为58.91%~116.80%，结果令人满意，说明该方法在实际水样和食品塑料接触材料分析中具有一定应用价值。

**表 3:** 矿泉水和泡面碗中4种溴代阻燃剂的测定（*n*=3）

Analyte	Spiked level/（μg/L）	Mineral water			Instant-noodle-bowl		
		Found/（μg/L）	Recovery/%	RSD/%	Found/（μg/L）	Recovery/%	RSD/%
TBBPA	0	ND	/	/	ND	/	/
	10	9.51	95.07	0.69	8.34	83.45	10.55
	20	23.36	116.80	6.20	22.79	113.94	6.61
PBB-2	0	ND	/	/	ND	/	/
	10	6.55	65.50	12.83	7.70	77.04	4.19
	20	17.11	85.54	3.59	17.26	86.31	5.20
PBB-15	0	ND	/	/	ND	/	/
	10	11.58	115.79	3.45	9.82	98.23	5.83
	20	22.43	112.16	3.43	21.97	109.87	6.86
BDE-47	0	ND	/	/	ND	/	/
	10	5.89	58.91	8.16	6.63	66.38	14.23
	20	14.29	71.48	4.87	15.05	75.26	5.18

ND： not detected； /： no value.

## 3 结论

本文制备了一种新型磁性碳凝胶材料MCA，并以此作为磁固相萃取材料，建立了磁固相萃取与高效液相色谱联用分析矿泉水和泡面碗中食品样品和食品接触材料中痕量溴代阻燃剂的新方法。与一般的磁固相萃取材料相比，MCA具有吸附性能好、富集倍数高的特点，可用于复杂基质中目标化合物的分离富集。此外，磁固相萃取可在外加磁场条件下进行快速固液分离，避免了繁琐的过滤或离心过程，操作简单便捷，增强了其实际应用价值。
